# A Chemically Defined Serum-Free Culture System for Spontaneous Human Mesenchymal Stem Cell Spheroid Formation

**DOI:** 10.1155/2020/1031985

**Published:** 2020-03-07

**Authors:** Yajun Zhao, E. Xiao, Wanqi Lv, Xian Dong, Linhai He, Yixiang Wang, Yi Zhang

**Affiliations:** ^1^Department of Oral and Maxillofacial Surgery, Peking University School and Hospital of Stomatology, Beijing 100081, China; ^2^Peking University Hospital of Stomatology First Clinical Division, Beijing 100081, China; ^3^Central Laboratory, Peking University School and Hospital of Stomatology, Beijing 100081, China

## Abstract

Mesenchymal stem cells (MSCs) possess promising potential in tissue engineering and regenerative medicine. Previous studies demonstrated that spheroid formation of MSCs exhibited improved stemness maintenance and therapeutic potential compared with monolayer culture. To date, various spheroid culture systems have been developed but most of them required low adhesion conditions or special equipment. In this study, we demonstrated that inoculation of dissociated MSCs in TeSR-E8 medium could induce self-assemble spheroid formation in conventional tissue culture polystyrene dishes. Compared with monolayer culture, adipose-derived stem cell (ADSC) spheroids enhanced the proliferation and osteogenic capability of ADSCs compared with monolayer culture. When reseeded in normal serum-containing medium, the expression level of stemness biomarkers was even higher in spheroid-derived ADSCs than monolayer culture. Importantly, spheroid ADSCs could effectively promote the M2 polarization of macrophages both *in vitro* and *in vivo*. After transplantation into mouse, spheroid ADSCs improved the survival rate and significantly decreased serum levels of proinflammatory factors IL-1*β* and TNF-*α* following LPS challenge. In summary, we developed a 3D spheroid culture system through TeSR-E8 medium without the involvement of low adhesion conditions and special equipment, which provided a practical and convenient method for spheroid formation of MSCs with great potential for stem cell clinical therapy.

## 1. Introduction

MSCs possess tremendous potential for tissue engineering and regenerative medicine due to their strong self-renewal, multipotent differentiation potential, and the immunomodulatory capacity [1]. Unlike embryonic stem cells (ESCs) or induced pluripotent stem cells (iPSCs), MSCs have almost no ethical issues or safety concerns [2], which is more beneficial for their clinical application. Although multiple sources of MSCs could be isolated from human tissues such as adipose tissue, bone marrow, dental pulp, or umbilical cord blood [3], large-scale expansion of MSCs is still essential to meet clinical needs. And the most important thing is how to maintain the stemness and the therapeutic potential of MSCs.

Traditionally, the standard method for *in vitro* expansion of MSCs is two-dimensional (2D) adherent culture. However, MSCs are usually subjected to impaired stemness and multipotency including a loss of proliferation rate and differentiation capacity during long-time, monolayer culture, which greatly reduced the therapeutic efficacy [4]. Thus, a variety of 3D culture systems have been developed to maintain the stemness properties of MSCs [5–7]. The approaches to 3D culture can be broadly categorized into two types: scaffold-free and scaffold-based [8]. Spheroid culture is the most commonly used scaffold-free 3D cell culture system, which has been widely applied in the field of tumorsphere [9], neurosphere [10], and embryoid body [11]. Many studies have demonstrated that MSCs cultured in 3D spheroids showed enhanced viability, stemness, and differentiation potential compared to monolayer cells [6, 12, 13]. Moreover, MSC spheroids have showed improved therapeutic efficacy in preclinical animal studies, such as bone and skin defect [14, 15], ischemic disease [16], and cardiovascular disorders [16, 17]. Thus, the application of 3D spheroid cell culture techniques is receiving an increased attention.

Numerous methods have been developed for the formation of MSC spheroids, which can be divided into static and dynamic culture system [18]. Static culture such as hanging drop [19] and low-attachment surfaces [17] has been widely employed for cell spheroid formation. However, these two methods are labor-intensive and not suitable for large-scale production of spheroids. Dynamic culture including spinner flasks and rotating wall vessels provides a feasible method for large-scale spheroid production, but the process is highly technical, which requires complex equipment to achieve suitable experimental setup and can cause shear stress damage on cell spheroids [20]. Therefore, an ideal spheroid culture system with simple operations and easy scale-up to mass production is still highly desired.

Normally, both MSCs and pluripotent stem cells are cultured in basal medium supplemented with serum of animal or human origin. However, the components in the serum have not been fully defined. Increasing evidence has indicated that commonly used serum such as fetal bovine serum (FBS) may contain endotoxin, mycoplasma, or viral contaminants [21], which restricts their wide use in clinical applications. Therefore, several defined serum-free mediums such as TeSR-E8 have been developed. TeSR-E8 medium is a highly chemically defined, xeno-free medium created by Chen et al. in the laboratory of James Thomson in 2011 [22]. As a feeder-free culture medium, TeSR-E8 contains only the essential components for cell culture and has been extensively tested and proved to maintain pluripotency in human ESCs [23] and iPSCs [24]. Previous study demonstrated that lipid deprivation of TeSR-E8 induced a naive-to-primed intermediate state of pluripotency in human pluripotent stem cells [25]. Thus, we hypothesized that TeSR-E8 could also help to maintain the stemness of MSCs, and tried to develop a novel method to maintain or enhance the stemness and therapeutic potential of human MSCs based on TeSR-E8 medium.

ADSCs represent an abundant source of MSCs due to their high extraction yield through low damage to donor area and have been widely used in 3D spheroid culture [7, 12, 26]. In the present study, we found that only using TeSR-E8 medium could induce self-assemble spheroid formation of human MSCs in tissue culture dishes within 3-5 days. The biological property of ADSC spheroids and their therapeutic effect in a mouse model of LPS-induced endotoxemia were also assessed. Overall, our study demonstrated that MSCs cultured in regular tissue culture dishes could spontaneously form 3D spheroids in a chemically defined serum-free culture condition without the involvement of low adhesion conditions and special equipment, which provided a convenient method for spheroid formation of MSCs with the maintenance of stemness and enhancement of their therapeutic potential.

## 2. Materials and Methods

### 2.1. Isolation and Identification of MSCs

All procedures were approved by the Ethics Committee of Peking University (PKUSSIRB-201948106), and all samples were collected with informed consents. Briefly, human adipose-derived stem cells (ADSCs) were obtained from the discarded subcutaneous adipose tissue after liposuction. Bone marrow mesenchymal stem cells (BMSCs) were obtained from trabecular bone of the mandibles following resection of redundant bones during third molar extraction or orthopedic surgery. Dental pulp stem cells (DPSCs) were obtained from the dental pulp of extracted wisdom teeth. Adipose tissue, bone tissue, and dental pulp were cut into small pieces and vibrated for 30 min in a solution containing dispase II (4 mg/mL) and collagenase I (3 mg/mL) at 37°C. The suspension was passed through a 70 *μ*m filter (BD Biosciences), collected by centrifugation at 200 g. Freshly isolated cells were cultured in *α*-modified Eagle's medium (*α*-MEM, Gibco) supplemented with 10% FBS (Gibco), 1% penicillin-streptomycin (Gibco), 2 mM l-glutamine (Gibco), and 10 mM l-ascorbic acid (Sigma-Aldrich), at 37°C in a humidified atmosphere with 5% CO_2_ for 24 h. Then, the cells were washed 3 times with PBS and continuously cultured. For detection of cell surface markers for MSCs, BD Stemflow Human MSC Analysis Kit (BD Biosciences) was used according to the manufacturer's instructions.

### 2.2. Spheroid Culture in TeSR-E8 Medium

For spheroid formation, MSCs at passages 3-5 grown as a monolayer were dissociated into single cells with 0.25% trypsin-EDTA solution (Gibco) and resuspended in TeSR-E8 medium supplemented with 1% penicillin-streptomycin. The initial cell density was approximately 50,000 cells/cm^2^. Spheroid formation was recorded by the Essen BioScience IncuCyte® Zoom Live-Cell Analysis System.

### 2.3. RNA Isolation and Quantitative Real-Time Polymerase Chain Reaction (qPCR) Analysis

Total RNA was extracted using the RaPure Total RNA Micro Kit (Magen) according to the manufacturer's instructions. Total RNA concentrations were measured using a NanoDrop 8000 Spectrophotometer (Thermo Scientific). Complementary DNA (cDNA) was prepared using the GoScript Reverse Transcription System (Promega). qPCR was performed with an ABI Prism 7500 sequence detection system (Applied Biosystems). The relative expression level of target genes was normalized to RPS18 and calculated using the 2^-*ΔΔ*Ct^ method. The primer sequences used in this study are listed in Table 1.

### 2.4. Multidifferentiation Assays

For osteogenic differentiation, monolayer and ADSC spheroids were induced in *α*-MEM supplemented with 10% FBS, 1% penicillin-streptomycin, 2 mM l-glutamine, 10 mM l-ascorbic acid, 100 nM dexamethasone (Sigma-Aldrich), and 10 mM *β*-glycerophosphate (Sigma-Aldrich). The osteogenic medium was changed every 3 days. At day 14, the expression of osteogenic markers, ALP and Runx2, was analyzed by qPCR and western blot.

For adipogenic differentiation, monolayer and ADSC spheroids were induced in *α*-MEM supplemented with 10% FBS, 1% penicillin-streptomycin, 2 mM l-glutamine, 10 mM l-ascorbic acid, 1 *μ*M dexamethasone, 0.5 mM 3-isobutyl-methylxanthin (IBMX, Sigma-Aldrich), 10 *μ*g/mL insulin (Sigma-Aldrich), and 60 *μ*M indomethacin (Sigma-Aldrich). The adipogenic medium was changed every 3 days. At day 7, the expression of adipogenic marker LPL was analyzed by qPCR and western blot.

### 2.5. Cell Proliferation Assay

Monolayer and spheroid-derived ADSCs were seeded at a density of 2,000 cells per well in 96-well plates. The cell number was assessed every other day till day 15 using the Cell Counting Kit-8 (Dojindo Laboratories) according to the manufacturer's instruction.

### 2.6. Cell Cycle Analysis

Cells were harvested by digestion and centrifugation. Then, the cell pellets were fixed with cold 70% ethanol for at least 30 min at 4°C. After washing twice in PBS, cells were collected by centrifugation and resuspended in PBS containing 0.1% Triton X-100 (Sigma-Aldrich) for 15 min. The cells were rinsed twice with PBS and treated with 50 *μ*g/mL RNase A (Sigma-Aldrich) for 30 min, then stained with 20 *μ*g/mL propidium iodide (PI, Sigma-Aldrich) for another 30 min. After staining, flow cytometry was performed with an Accuri C6 flow cytometer.

### 2.7. Live/Dead Assay for Cell Viability

Live/Dead Viability/Cytotoxicity Assay Kit (Keygen) was used to visualize the viable and dead cells in ADSC spheroids. The viability of spheroid cells was determined by staining with calcein AM to label the living cells and with PI to label the dead cells. Spheroids were incubated in PBS containing 2 *μ*M calcein AM and 8 *μ*M PI for 30-45 min. After washing twice with PBS, the cells were observed using a fluorescence microscope.

### 2.8. Immunofluorescence

Spheroid staining was performed by embedding them in optimum cutting temperature (OCT) compound for the preparation of cryostat sections. For immunofluorescence staining, specimens were immersed in PBS containing 0.1% Triton X-100 and 1% normal serum for 30 min at room temperature. Samples were then incubated overnight at 4°C with rabbit anti-Ki67 (1 : 100, Abcam). After incubation with primary antibody, cells were washed with PBS and then incubated with FITC-conjugated goat anti-rabbit IgG for 1 h at room temperature. Nuclear DNA was dyed with DAPI. Images were captured and analyzed with a fluorescent microscope.

### 2.9. Western Blot Assay

Cells were harvested, washed with PBS, and lysed in RIPA buffer (Applygen) containing protease inhibitor (Thermo Scientific). Primary antibodies against ALP (Abcam), RUNX2 (Cell Signaling Technology), LPL (Santa Cruz), PPAR-*γ* (Santa Cruz), and GAPDH (Zhongshan Jinqiao) were diluted 1 : 1,000 and incubated with the membranes at 4°C overnight. Horseradish peroxidase-conjugated anti-rabbit or anti-mouse secondary antibodies (Zhongshan Jinqiao) were diluted 1 : 10,000 and incubated with the membranes at room temperature for 1 h. The membranes were then visualized using an ECL kit (Proteintech). Band intensities were quantified using ImageJ software. The background was subtracted, and the signal of each target band was normalized to that of the GAPDH band.

### 2.10. Macrophage Inflammatory Assay

Conditioned medium (CM) was collected from monolayer ADSCs and ADSC spheroid cultures after 1 day and subjected to filtration (0.22 *μ*m filter). RAW 264.7 mouse macrophages were cultured in high-glucose DMEM containing 10% FBS and 1% penicillin-streptomycin. For the inflammatory assay, RAW 264.7 cells were seeded at 25,000 cells/cm^2^ onto 6-well culture plates. After 1 day, monolayer-CM and spheroid-CM were added to the macrophage culture medium at 1 : 3 dilution, respectively. After 48 h culture, mouse macrophages were washed with PBS and harvested for RNA to quantify Arg-1 and IL-10 expression levels by qPCR. For flow cytometry analysis, M2 macrophages were identified as CD206-positive cells. The cells were stained with PE-conjugated anti-mouse CD206 (BioLegend) according to the manufacturer's guide. The percentage of cells staining positive for CD206 was determined by comparing the test samples to macrophages labeled with isotype control antibody. After staining, flow cytometry was performed with a BD LSR II flow cytometer.

### 2.11. Animal Model and Cell Transplantation

Experimental procedures involving animals followed institutionally approved protocols for animal research at Peking University (LA2019324). Adult male C57BL/6 mice (6-8 weeks old) were purchased from Beijing Vital River Laboratory Animal Technology Co., Ltd., (Beijing, China). Mice were randomly divided into three groups: (i) LPS challenge (control group); (ii) LPS+monolayer ADSCs (monolayer ADSC group); and (iii) LPS+ADSC spheroids (ADSC spheroid group). Endotoxemia was induced by i.p. administration of 25 mg or 50 mg LPS per kg of mouse weight (From *Escherichia coli* serotype 055:B5, Sigma-Aldrich). At 1 h after LPS injection, mice received an i.p. injection of PBS or ADSCs in PBS (2 × 10^6^ cells/mouse). The monolayer ADSC group received injection of ADSCs in the form of dissociated cells and ADSC spheroid group in the form of cell spheroids. Survival of mice after LPS challenge was monitored for 48 h. Serum and spleen samples were collected at 6 h after LPS (50 mg/kg) challenge and used for cytokine measurement and flow cytometry analysis.

### 2.12. Enzyme-Linked Immunosorbent Assay (ELISA)

Serum samples were obtained following blood separation by centrifugation at 6,500 g for 40 min at 4°C and were stored at -80°C until analysis. Serum levels of IL-1*β* and TNF-*α* were determined using an ELISA with mouse-specific kits (Neobioscience) following the manufacturer's instructions.

### 2.13. Detection of Tregs and Macrophage Polarization by Flow Cytometry

Spleens were isolated from the mice, ground, and passed through a 70 *μ*m cell strainer (Falcon) to get single-cell suspension. The cells were stained with FITC-conjugated anti-mouse CD4 (ebioscience), APC-conjugated anti-mouse CD25 (ebioscience), PE-conjugated anti-mouse Foxp3 (ebioscience), PerCP-Cy5.5-conjugated anti-mouse F4/80 (ebioscience), APC/Cy7-conjugated anti-mouse CD11b (BioLegend), FITC-conjugated anti-mouse CD86 (BioLegend), and PE-conjugated anti-mouse CD206 (BioLegend) according to the manufacturer's guide. After staining, flow cytometry was performed with a BD LSR II flow cytometer. CD4^+^CD25^+^Foxp3^+^ cells were marked as Treg cells. CD11b^+^F4/80^+^CD86^+^ cells were marked as M1 macrophages. CD11b^+^F4/80^+^CD206^+^ cells were marked as M2 macrophages.

### 2.14. Statistical Analysis

Statistical analysis was performed by one-way analysis of variance followed by Bonferroni's multiple-comparison post hoc test and unpaired Student's *t*-test. Survival data are presented in Kaplan-Meier curves, and statistical significance was assessed using the log rank test. A *P* value of less than 0.05 was considered statistically significant.

## 3. Results

### 3.1. Spontaneous Spheroid Formation of MSCs in TeSR-E8 Medium

**Figure 1:**
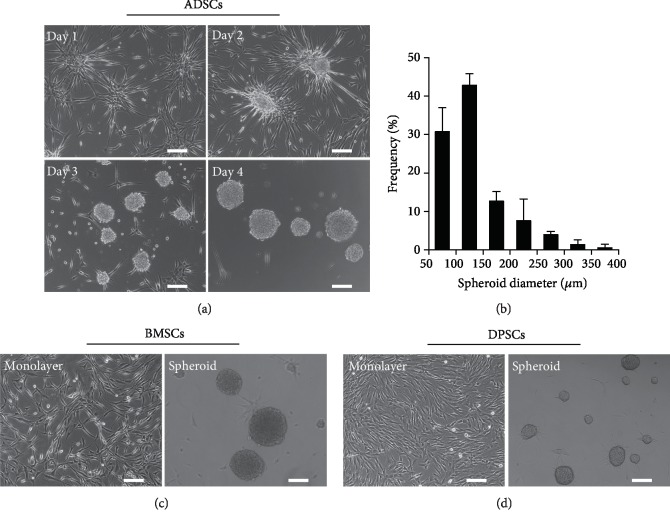
MSCs spontaneously form spheroids in TeSR-E8 medium on tissue culture dishes. (a) Phase contrast images showing the time-course of ADSC spheroid formation over 4 days. Scale bar: 150 *μ*m. (b) Size distribution of ADSC spheroids on day 4 postculture. (c, d) Morphology of monolayer and spheroid BMSCs and DPSCs. Scale bar: 150 *μ*m.

The morphology of cells exposed to TeSR-E8 medium in conventional tissue culture dishes is shown in Figure 1(a). Single cells dissociated from the monolayer ADSCs attached and spread on stiff substrates to form a net-like structure and then gradually retracted their pseudopodia before aggregating into a 3D sphere-like structure. After that, the multicellular spheroids gradually detached from the bottom of the culture dish and survived in a suspension state. At the same time, adjacent spheroids could coalesce to form larger ones. The dynamic process of spheroid formation was recorded by the IncuCyte® Zoom Live-Cell Analysis System, and typical real-time images were included in [Supplementary-material supplementary-material-1]. The average diameters of multicellular spheroids were 133.77 ± 57.12 *μ*m, mostly between 50 and 200 *μ*m after 4 days of culture (Figure 1(b)). To verify whether MSCs from other sources have the same characteristics as ADSCs, two types of MSCs, BMSCs and DPSCs, were also cultured in TeSR-E8 medium. Results showed that compared with the adherent state in serum-containing medium, spontaneous spheroid formation was also observed in BMSCs and DPSCs that were cultured in TeSR-E8 medium (Figures 1(c) and 1(d)). These data indicated that spontaneous spheroid formation of cells cultured in TeSR-E8 medium was a universal phenomenon among human MSCs.

### 3.2. Expression of Surface-Specific Antigens and Pluripotent Markers of Spheroid ASCs

**Figure 2:**
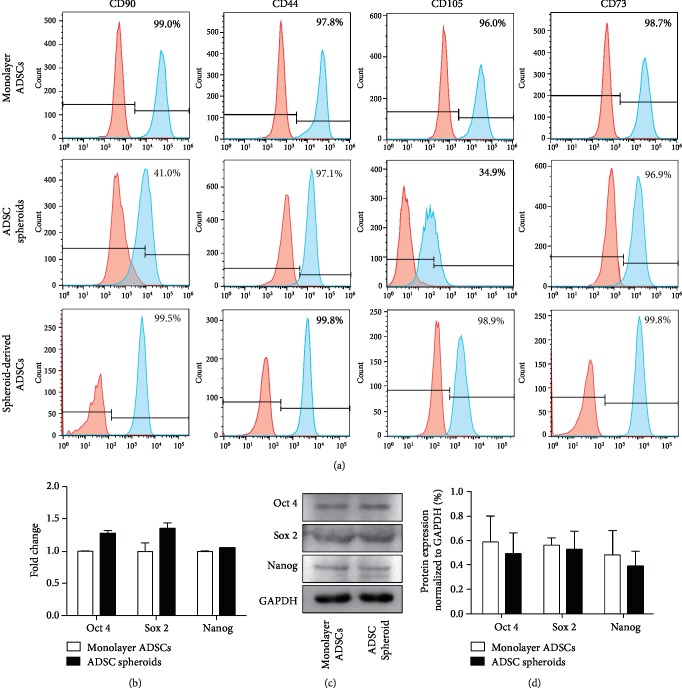
Phenotypic characterization and expression of pluripotent markers in spheroid and monolayer ADSCs. (a) The expression level of MSC surface-specific antigens was determined by flow cytometry after 4 days of monolayer and spheroid culture. Spheroid-derived ADSCs were referred to monolayer ADSCs migrated from cell spheroids cultured in serum-containing medium. (b) qPCR measurements for stemness marker genes (Oct 4, Sox 2, and Nanog) in spheroid and monolayer ADSCs. Data are displayed as mean ± SEM. (c) Western blot analysis of the expression of Oct 4, Sox 2, and Nanog in ADSC spheroids and monolayer ADSCs. (d) Quantification of protein levels compared to GAPDH control. Data are displayed as mean ± SEM.

We analyzed the expression of MSC surface markers (CD90, CD44, CD105, and CD73) in monolayer ADSCs and ADSC spheroids by flow cytometry on day 4 postculture (Figure 2(a)). All the above cell surface antigens were positive (>95%) in monolayer ADSCs. Compared with monolayer culture, ADSCs within the spheroids showed a dramatic decrease in the expression of CD90 and CD105, along with a slight decrease in the expression of CD44 and CD73. After cell spheroids seeded back to normal serum-containing medium and turned into monolayer growth (referred as spheroid-derived ADSCs), cells were collected again for detection of surface antigens. Results showed that when cultured in serum-containing medium, the cells derived from spheroids increased the expression level of CD90 and CD105 compared with ADSC spheroids, and the expression of all detected biomarkers restored to a similar level of those in monolayer ADSCs.

The relative mRNA expression of pluripotent markers Oct 4, Sox 2, and Nanog was analyzed by qPCR. As shown in Figure 2(b), the expression level of Oct 4, Sox 2, and Nanog in ADSC spheroids was comparable with that in monolayer ADSCs. There was no significant difference between groups. Western blot analysis (Figures 2(c) and 2(d)) also showed that in comparison with monolayer ADSCs, ADSC spheroids did not exhibit significant decrease in the expression of pluripotent markers. These data indicated that the expression of stemness markers was maintained in ADSC spheroids induced by TeSR-E8 medium culture.

### 3.3. Viability and Proliferation Properties of ADSC Spheroids

**Figure 3:**
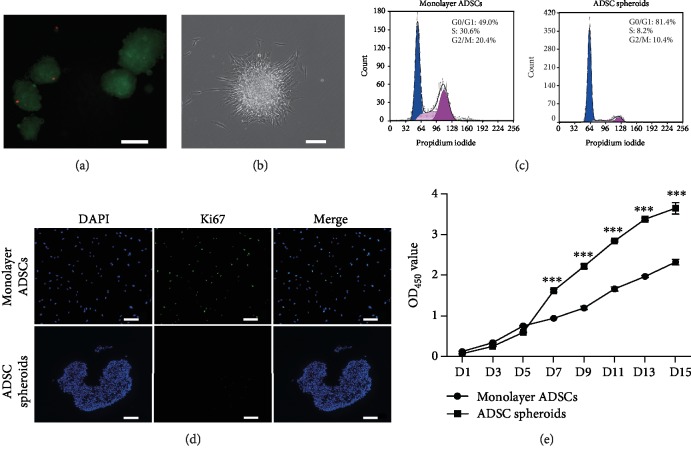
The viability and proliferative capacity of ADSC spheroids. (a) Fluorescence microscopic images of live/dead staining on day 4 postculture. Live cells within spheroids were stained with calcein AM (green), and dead cells were stained with PI (red). Scale bar: 100 *μ*m. (b) Morphology of ADSC spheroids after replacing TeSR-E8 with serum-containing medium. Scale bar: 150 *μ*m. (c) Cell cycle analysis via flow cytometry of monolayer ADSCs and ADSC spheroids. (d) Representative immunostaining of proliferation marker Ki67 in spheroid and monolayer ADSC after 4 days of culture. All nuclei were stained with DAPI (blue). Scale bar: 150 *μ*m. (e) Cell proliferation analysis using the CCK-8 assay. Data are displayed as mean ± SEM.^∗∗∗^*P* < 0.001 vs. monolayer ADSCs.

The viability of spheroid cells was assessed using a live/dead assay. As shown in Figure 3(a), most cells were viable despite diffusion limitations for oxygen and nutrition at the core of spheroids. After replacing TeSR-E8 with conventional serum-containing medium, suspended cell spheroids adhered to the culture dish again and cells spread out from the edge to restore monolayer growth (Figure 3(b)).

To verify the proliferation state of ADSC spheroids, the cell cycle distribution was determined by analyzing DNA content of cells labeled with PI. Flow cytometry analysis showed that compared with monolayer control, ADSC spheroids had higher ratios of cells at G0/G1 phases and lower ratios of cells at S phase and G2/M phases (Figure 3(c)). Furthermore, we detected the protein level of Ki-67, a proliferation marker, via an immunofluorescence assay. As shown in Figure 3(d), there was a decline of Ki-67 protein level expressed in the ADSC spheroids, indicating that cells in the spheroids were negatively proliferating.

To evaluate the proliferative potential of ADSC spheroids in serum-containing medium, ADSC spheroids were dissociated into single cells and reseeded in tissue culture dishes. CCK-8 showed that compared with monolayer ADSCs, cells from ADSC spheroids exhibited stronger proliferative capacity; statistical differences were observed from day 7 to day 15 (Figure 3(e)). These data suggested that the proliferative capacity of ADSCs within spheroids was suppressed in TeSR-E8 medium but was further enhanced after exposure to serum.

### 3.4. Differentiation Property of ADSC Spheroids

**Figure 4:**
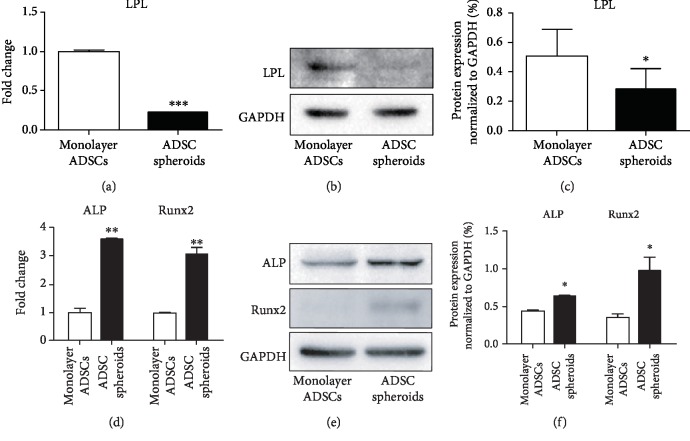
Differentiation capabilities of monolayer ADSCs and ADSC spheroids after 7 days of adipogenic induction and 14 days of osteogenic induction. (a, d) qPCR measurements for adipogenic marker LPL and osteogenic markers ALP and Runx2 in monolayer ADSCs and ADSC spheroids. Data are displayed as mean ± SEM.^∗∗^*P* < 0.01, ^∗∗∗^*P* < 0.001 vs. monolayer ADSCs. (b, e) Western blot analysis of the expression of LPL, ALP, and Runx2 in monolayer ADSCs and ADSC spheroids. (c, f) Quantification of protein levels compared to GAPDH control. Data are displayed as mean ± SEM. ^∗^*P* < 0.05 vs. monolayer ADSCs.

To investigate the effects of spheroid formation on the differentiation capability of ADSCs, cell spheroids were collected and suspended in the bottom of a 15 mL centrifuge tube and induced by the same adipogenic and osteogenic induction media as monolayer ADSCs. Cell spheroids remained spherical during induction. The mRNA and protein expressions of adipogenic marker gene lipoprotein lipase (LPL) and osteogenic marker genes alkaline phosphatase (ALP) and runt-related transcription factor 2 (Runx2) were evaluated to identify the adipogenic and osteogenic differentiation, respectively. As shown in Figure 4(a), qPCR analysis showed that the adipocyte-specific mRNA expression level of LPL was significantly decreased in ADSC spheroids compared with monolayer ADSCs after 7 days of adipogenic induction. Western blot analysis also showed less expression of LPL in ADSC spheroids compared to monolayer culture (Figures 4(b) and 4(c)). Conversely, ADSC spheroids exhibited higher mRNA levels of osteogenic markers ALP and Runx2 after 14 days of osteogenic induction (Figure 4(d)). Increased protein levels of ALP and Runx2 in ADSC spheroids were also detected by western blot analysis (Figures 4(e) and 4(f)). These results demonstrated that ADSCs within spheroids possessed enhanced osteogenic capability along with decreased adipogenic capability.

### 3.5. ADSC Spheroids Promote Polarization of Macrophages into M2 Phenotype More Effectively In Vitro

**Figure 5:**
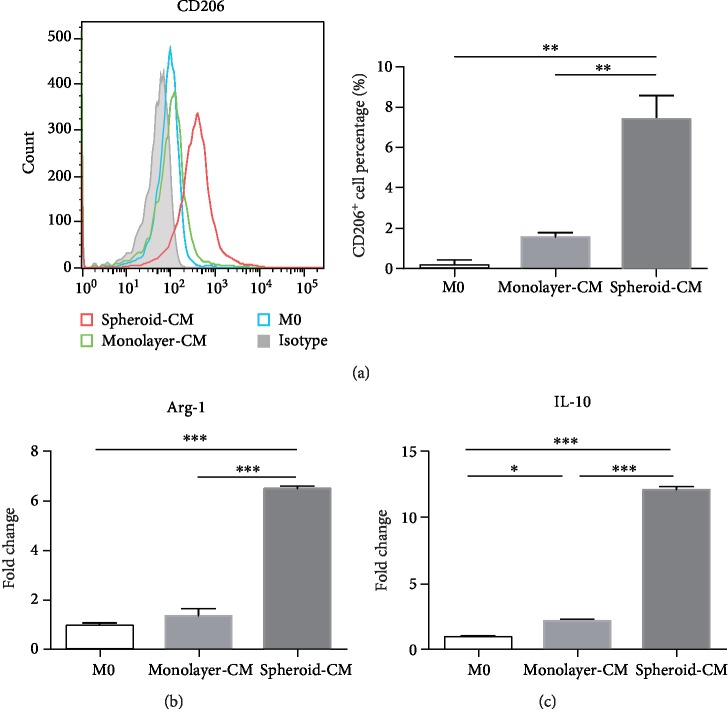
ADSC spheroids promote polarization of macrophages into M2 phenotype more effectively *in vitro*. (a) The percentage of CD206-positive macrophages was determined by flow cytometry. Data are displayed as mean ± SEM. ^∗∗^*P* < 0.01. (b, c) qPCR measurements for M2 macrophage markers Arg-1 and IL-10. Data are displayed as mean ± SEM. ^∗^*P* < 0.05, ^∗∗∗^*P* < 0.001.

Previous study has demonstrated that MSCs could convert macrophages into an anti-inflammatory M2 phenotype by secreting soluble factors [27]. To compare the ability of spheroid and monolayer ADSCs in inducing macrophages' M2 polarization, ADSC spheroid- and monolayer ADSC-conditioned mediums (CM) were collected and added to macrophage culture medium in a ratio of 1 : 3. After 48 h, flow cytometry analysis showed that spheroid-CM significantly increased the number of CD206^+^ macrophages compared with unstimulated macrophages and monolayer-CM, with 29.64-fold and 4.72-fold increase, respectively (Figure 5(a)). In addition, qPCR analysis showed that compared with monolayer-CM, spheroid-CM was more effective in promoting macrophages mRNA expression of M2 markers such as Arg-1 and IL-10. Compared to M0 macrophages, the expression level of Arg-1 increased by 1.35 ± 0.21 and 6.50 ± 0.07 times in the monolayer-CM and spheroid-CM group, respectively (Figure 5(b)). And the expression level of IL-10 increased by 2.20 ± 0.10 and 12.10 ± 0.18 times, respectively (Figure 5(c)). These results suggested that ADSC spheroids were more effective in converting macrophages into M2 phenotype through a paracrine manner.

### 3.6. ADSC Spheroids Exhibit Stronger Therapeutic Potential in LPS-Induced Endotoxemia

**Figure 6:**
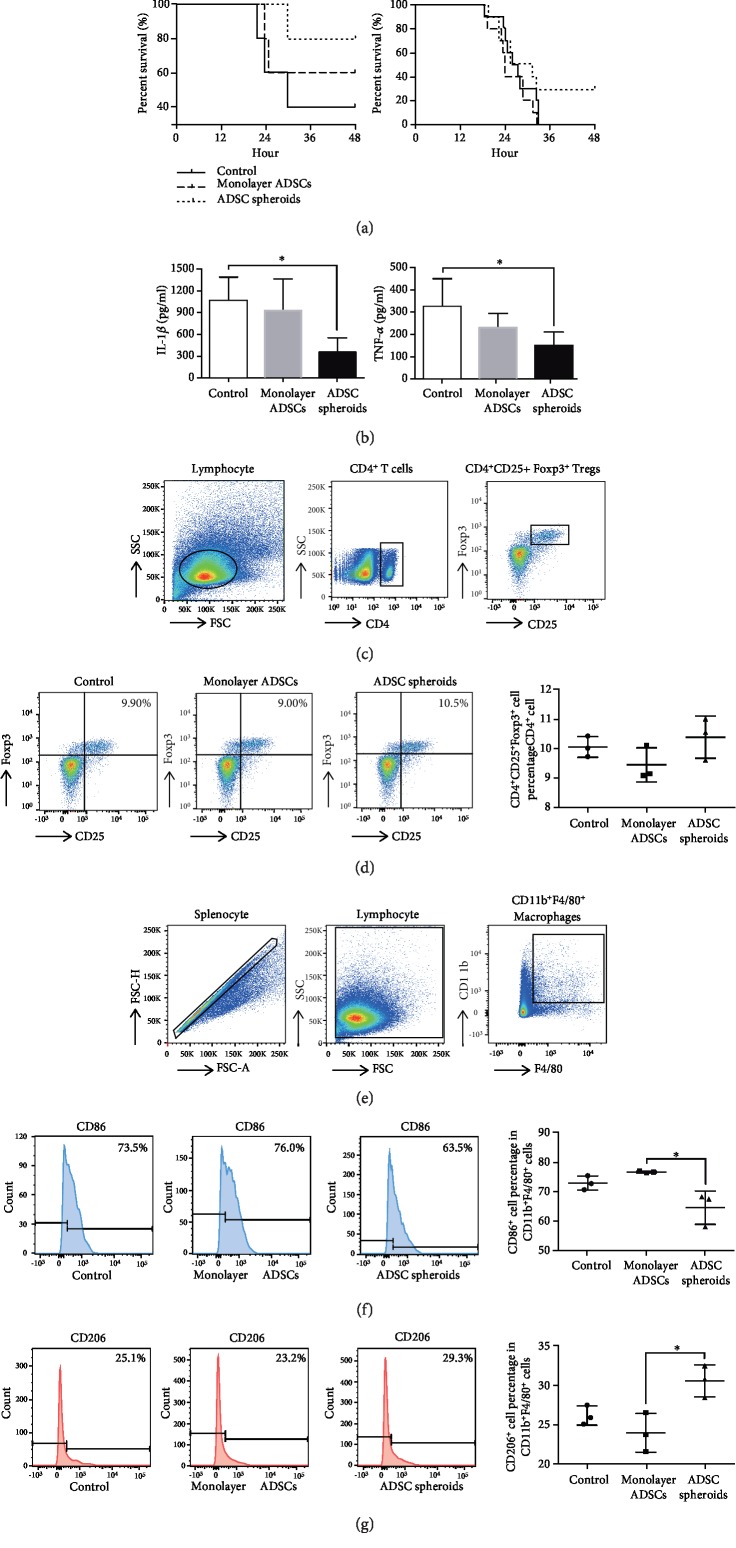
Therapeutic potential of ADSC spheroids in a mouse model of LPS-induced endotoxemia. (a) Survival rates of mice with LPS-induced endotoxemia following monolayer ADSCs and ADSC spheroid treatment within 48 h. Administration dosage of LPS was 25 mg/kg (*n* = 5 mice/group) and 50 mg/kg (*n* = 10 mice/group). (b) Cytokine levels were determined by ELISA in protein extracts from blood serum collected at 6 hours after LPS injection (50 mg/kg, *n* = 3-5 mice/group). ^∗^*P* < 0.05 vs. the control group. (c) Gating strategy for identification of Tregs with flow cytometry. (d) The percentages of CD4^+^CD25^+^Foxp3^+^ cells in the CD4 population of splenocytes isolated at 6 h after LPS injection (50 mg/kg, *n* = 3 mice/group). (e) Gating strategy for identification of macrophages with flow cytometry. (f, g) The percentages of CD86^+^ (M1 macrophage biomarker) or CD206^+^ (M2 macrophage biomarker) cells in CD11b^+^F4/80^+^ population (total macrophages) of splenocytes isolated at 6 h after LPS injection (50 mg/kg, *n* = 3 mice/group). ^∗^*P* < 0.05 vs. the monolayer ADSC group.

We used a mouse model of LPS-induced endotoxemia to assess the therapeutic effects of ADSC spheroids *in vivo*. As shown in Figure 6(a), when the stimulation dosage of LPS was 25 mg/kg (*n* = 5/group), survival analysis showed that 80% mice survived in the ADSC spheroid group, 60% mice survived in the monolayer ADSC group, and only 40% mice survived in the control group within 48 h. When the administration dosage of LPS was increased to 50 mg/kg (*n* = 10/group), all the mice in the control and monolayer ADSC group succumbed to mortality, with 30% mice that survived in the ADSC spheroid group.

To examine the serum levels of proinflammatory factors IL-1*β* and TNF-*α*, ELISA was performed at 6 h after LPS exposure. The serum levels of IL-1*β* and TNF-*α* were significantly lower in the ADSC spheroid group compared with the control group. However, there was no significant difference in the monolayer ADSC group compared with the control group (Figure 6(b)). The observed downregulated proinflammatory factors prompted us to investigate the impact of ADSC spheroids on the inflammatory cells including the Tregs and activated macrophages *in vivo*. Flow cytometry analysis of the spleen showed that there was no significant difference of CD4^+^CD25^+^Foxp3^+^ Treg percentage in CD4^+^ cells among the three groups (Figures 6(c) and 6(d)). However, compared with monolayer ADSCs, ADSC spheroids significantly decreased the CD86^+^ M1 macrophages (Figures 6(e) and 6(f)) and increased the CD206^+^ M2 macrophages in the splenocytes of the mice (Figures 6(e) and 6(g)).

## 4. Discussion

Adult stem cells such as MSCs have shown broad and promising application prospects. However, maintaining the stemness properties and enhancing the therapeutic potential of MSCs during traditional adherent culture are still challenging. In order to overcome the defects of *in vitro* monolayer expansion, a great deal of research has paid attention on 3D spheroid culture for the improvement of biological properties over the past decade [18, 28]. Among various spheroid culture systems, most of them required low adhesion conditions (low attachment culture dishes or chitosan membranes) or special equipment (spinner flasks or rotating wall vessels), which also limited large-scale application in clinical trials. In the present study, we have developed a self-assemble 3D spheroid culture system through TeSR-E8 medium without the involvement of exogenous components and special equipment. In this way, three types of human MSCs spontaneously migrated and aggregated into a spherical structure in conventional tissue culture dishes.

Despite the promising potential that MSC spheroids have in clinical application, research on the underlying mechanisms that trigger and mediate the formation process of multicellular spheroids is still limited. Based on previous studies, the following mechanisms have been proposed. In low attachment conditions, integrin-extracellular matrix binding encourages initial loose cell aggregate, and homophilic cadherin (a type of Ca^2+^-dependent transmembrane adhesion molecular) binding promotes strong spheroid compaction [29]. On chitosan membranes, the calcium-binding capacity of chitosan may elevate intracellular calcium level and increase the expression of cadherin, which changes the cell-substrate and cell-cell interactions to modulate the dynamics of spheroid formation [30]. In the present study, the internal mechanism of spontaneous spheroid formation in TeSR-E8 medium is not yet known. TeSR-E8 is a highly chemically defined, xeno-free medium that contains only eight components, which consist of insulin, selenium, transferrin, NaHCO_3_, l-ascorbic acid, bFGF, TGF*β* (or Nodal), and DMEM/F12 [22]. Spheroid formation through TeSR-E8 medium avoids the disadvantages of using serum-containing medium. More importantly, it will be more practical to define the critical component in TeSR-E8 medium that promotes spheroid formation and investigate the underlying mechanisms in the future.

Large-scale expansion of MSCs with stemness maintenance is vital for effective stem cell transplantation and therapeutic potential [6]. However, continuous *in vitro* passaging declined the expression of several pluripotent markers and the stemness properties such as proliferation and differentiation [31]. Thus, it is necessary to develop techniques to preserve MSCs stemness *in vitro*. Our new finding showed that spheroid formation of ADSCs in TeSR-E8 had a potential to maintain the expression of pluripotent markers and enhance the proliferative and osteogenic capabilities. The expression level of surface-specific antigens of spheroid-derived ADSCs was even higher compared with monolayer culture. Previous study has demonstrated that lipid deprivation of TeSR-E8 induced a naive-to-primed intermediate state of pluripotency via endogenous ERK inhibition in human pluripotent stem cells [25]. Thus, ADSCs within spheroids in TeSR-E8 medium may also be induced to dedifferentiate into a more primitive state as pluripotent stem cells.

We also observed that the surface epitopes expressed by ADSC spheroids were quite different from those of monolayer ADSCs. CD90 and CD105 showed a dramatic decrease in ADSC spheroids, which was consistent with spheroid formation via a spinner flask [20], rotating wall vessel bioreactor [20], or chitosan films [6]. These changes observed in spheroid culture were reversible after cell spheroid exposure to serum and growing as monolayers. However, the underlying mechanisms have not yet been elucidated. Both of CD90 and CD105 were demonstrated to be associated with cytoskeletal organization regulation [32, 33]. During spheroid formation, cytoskeleton reorganization was accompanied by reduced gene expression of *β*-actin [34] and cytoskeleton tension [35]. Thus, changes of surface-specific antigen expression and cytoskeleton reorganization during spheroid formation may interact with each other to determine the biological characteristics of ADSC spheroids. Previous study has demonstrated that a reduction in CD90 expression could enhance the osteogenic differentiation of MSCs [36]. Our study showed that 3D spheroid of ADSCs could reduce CD90 expression and enhance the osteogenic differentiation, which was in line with the above reference [36].

The conventional 2D culture often does not adequately imitate the status of living cells *in vivo* [18]. Changes in cell morphology, gene expression, etc. led to drastic difference of biological characteristics such as viability, differentiation, and cytokine secretion between MSCs cultured in spheroid and in monolayer [18, 37, 38]. Hence, the impact of spheroid formation on cell behaviors still needs further investigation. Previous study has showed a decrease in the S phase of spheroid BMSCs formed by hanging drop compared with the monolayer control [13]. In the present study, high level of cell cycle arrest was also observed in ADSC spheroids. When exposed to serum-containing medium, cells from spheroids exhibited even stronger proliferation capability. Thus, serum deprivation may have been a contributing factor for cell cycle arrest. On the other hand, ADSC spheroids exhibited improved osteogenic and decreased adipogenic property compared with monolayer ADSCs. Multiple factors regulate the balance between adipogenic and osteogenic differentiation of MSCs, including chemical, physical, and biological factors [39]. During spheroid formation, cell shape, mechanical forces, extracellular matrix, and cell metabolism (a hypoxia environment) changed a lot from monolayer culture [18, 40], which can break the adipoosteogenic balance of ADSCs.

MSC spheroids have showed improved therapeutic efficacy in several disease models. Grafting stem cells as 3D spheroids enhances cell survival and angiogenesis in ischemic tissue and protects limb muscle from necrotic damage [16]. MSC spheroids also exhibit enhanced osteoregenerative potential that promotes the healing of rat calvarial defects [14]. However, the effect of MSC spheroids on endotoxemia has not been reported. We explored the therapeutic potential of ADSC spheroids in a mouse model of LPS-induced endotoxemia. ADSC spheroid administration led to the highest survival rate among the three groups. Furthermore, the ADSC spheroid group also showed a downregulated serum level of proinflammatory factors and upregulated M2 macrophage polarization among splenocytes. Although the accumulated survival rate had no significant difference among the three groups, these data also predicted a better therapeutic efficacy of ADSC spheroids. LPS-induced endotoxemia is an acute inflammatory model with the characteristics of severe attack and high fatality rate. ADSC spheroids may exhibit better therapeutic potential in chronic diseases than acute illness.

## 5. Conclusions

In conclusion, we developed a simple and practical method to generate MSC spheroids in a chemically defined TeSR-E8 medium in conventional tissue culture polystyrene dishes. Spheroid formation through this method maintained the stemness and enhanced the therapeutic efficacy in the mouse model of LPS-induced endotoxemia, which possesses great potential for clinical applications without the involvement of exogenous components, low adhesion conditions, and special equipment.

## Figures and Tables

**Table 1:** Primers used in real-time polymerase chain reaction gene expression analysis.

Gene	Primer sequence	
RPS18	Forward	GCGGCGGAAAATAGCCTTTG
	Reverse	GATCACACGTTCCACCTCATC

Oct 4	Forward	CTTGAATCCCGAATGGAAAGGG
	Reverse	GTGTATATCCCAGGGTGATCCTC

Sox 2	Forward	CATGAAGGAGCACCCGGATT
	Reverse	GTTCATGTGCGCGTAACTGT

Nanog	Forward	AATGGTGTGACGCAGGGATG
	Reverse	TGCACCAGGTCTGAGTGTTC

LPL	Forward	TCATTCCCGGAGTAGCAGAGT
	Reverse	GGCCACAAGTTTTGGCACC

ALP	Forward	AACATCAGGGACATTGACGTG
	Reverse	GTATCTCGGTTTGAAGCTCTTCC

Runx2	Forward	TGGTTACTGTCATGGCGGGTA
	Reverse	TCTCAGATCGTTGAACCTTGCTA

Arg-1	Forward	CTCCAAGCCAAAGTCCTTAGAG
	Reverse	AGGAGCTGTCATTAGGGACATC

IL-10	Forward	CTTACTGACTGGCATGAGGATCA
	Reverse	GCAGCTCTAGGAGCATGTGG

## Data Availability

The data associated with in vitro induction and in vivo transplantation used to support the findings of this study, including the results and the methods & materials, are included within the article or the supplementary materials.

## Supplementary Materials

Supplementary Materials Video 1: the dynamic process of ADSC spheroid formation in TeSR-E8 medium. The video was obtained by the IncuCyte Live-Cell Analysis System.
