# Corrigendum to “A Chemically Defined Serum-Free Culture System for Spontaneous Human Mesenchymal Stem Cell Spheroid Formation”

**DOI:** 10.1155/2020/8241367

**Published:** 2020-09-01

**Authors:** Yajun Zhao, E. Xiao, Wanqi Lv, Xian Dong, Linhai He, Yixiang Wang, Yi Zhang

**Affiliations:** ^1^Department of Oral and Maxillofacial Surgery, Peking University School and Hospital of Stomatology, Beijing 100081, China; ^2^Peking University Hospital of Stomatology First Clinical Division, Beijing 100081, China; ^3^Central Laboratory, Peking University School and Hospital of Stomatology, Beijing 100081, China

In the article titled “A Chemically Defined Serum-Free Culture System for Spontaneous Human Mesenchymal Stem Cell Spheroid Formation” [1], there was an error in the Acknowledgments section. The corrected section appears below:
